# Prevention of early-onset cardiomyopathy in *Dmd* exon 52–54 deletion mice by CRISPR-Cas9-mediated exon skipping

**DOI:** 10.1016/j.omtm.2023.07.004

**Published:** 2023-07-17

**Authors:** Matthew Rok, Tatianna Wai Ying Wong, Eleonora Maino, Abdalla Ahmed, Grace Yang, Elzbieta Hyatt, Kyle Lindsay, Sina Fatehi, Ryan Marks, Paul Delgado-Olguín, Evgueni A. Ivakine, Ronald D. Cohn

**Affiliations:** 1 Program in Genetics and Genome Biology, The Hospital for Sick Children Research Institute, Toronto, ON, Canada; 2 Department of Molecular Genetics, University of Toronto, Toronto, ON, Canada; 3 Institute of Medical Science, University of Toronto, Toronto, ON, Canada; 4 Department of Pediatrics, The Hospital for Sick Children, Toronto, ON, Canada; 5 Department of Physiology, University of Toronto, Toronto, ON, Canada; 6 Department of Translational Medicine, The Hospital for Sick Children, Toronto, ON, Canada; 7 Heart & Stroke Richard Lewar Centre of Excellence, Toronto, ON, Canada; 8 Department of Biochemistry & Biomedical Sciences, McMaster University, Hamilton, ON, Canada

**Keywords:** CRISPR, Duchenne muscular dystrophy, exon skipping, genome editing, *in vivo*, cardiomyopathy, AAV, dystrophin, heart, systemic

## Abstract

Duchenne muscular dystrophy (DMD) is a disease with a life-threatening trajectory resulting from mutations in the dystrophin gene, leading to degeneration of skeletal muscle and fibrosis of cardiac muscle. The overwhelming majority of mutations are multiexonic deletions. We previously established a dystrophic mouse model with deletion of exons 52–54 in *Dmd* that develops an early-onset cardiac phenotype similar to DMD patients. Here we employed CRISPR-Cas9 delivered intravenously by adeno-associated virus (AAV) vectors to restore functional dystrophin expression via excision or skipping of exon 55. Exon skipping with a solitary guide significantly improved editing outcomes and dystrophin recovery over dual guide excision. Some improvements to genomic and transcript editing levels were observed when the guide dose was enhanced, but dystrophin restoration did not improve considerably. Editing and dystrophin recovery were restricted primarily to cardiac tissue. Remarkably, our exon skipping approach completely prevented onset of the cardiac phenotype in treated mice up to 12 weeks. Thus, our results demonstrate that intravenous delivery of a single-cut CRISPR-Cas9-mediated exon skipping therapy can prevent heart dysfunction in DMD *in vivo*.

## Graphical abstract

**Figure:**
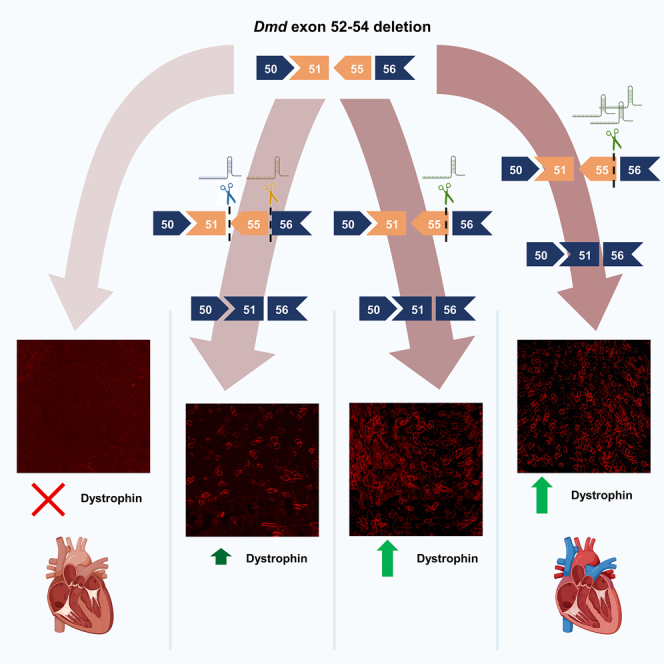

## Teaser

Rok and colleagues treated a novel *Dmd* deletion mouse with three unique CRISPR-Cas9 exon skipping strategies delivered by a therapeutically translatable route. Single-cut exon skipping was shown to be effective primarily at restoring dystrophin in the heart, completely preventing onset of cardiomyopathy in treated mice.

## Introduction

Duchenne muscular dystrophy (DMD) is the most prevalent X-linked pediatric neuromuscular disease with an incidence of around 1 in 5,000 males.^1^^,^^2^^,^^3^ DMD exerts a high disease burden, with those afflicted experiencing progressive, systemic muscle wasting early in life. Throughout childhood, independent ambulation is gradually lost, with upper extremity weakness closely following.^2^^,^^4^^,^^5^^,^^6^^,^^7^ Skeletal and cardiac muscle are affected, impairing the diaphragm and heart from carrying out their essential functions. Cardiorespiratory failure typically emerges in a patient’s mid-teens to late twenties, although advancements in non-invasive ventilation have enabled DMD patients to live, on average, into their thirties and forties.^2^^,^^4^^,^^5^^,^^6^^,^^7^ In turn, heart dysfunction resulting from cardiomyopathy is now the leading cause of death among DMD patients because current treatment methods are of limited effectiveness over time.^7^^,^^8^^,^^9^^,^^10^

Dystrophin is a subsarcolemmal protein encoded by the *DMD* gene, which is essential to the association and integrity of linkages of intracellular cytoskeletal elements to the sarcolemma and extracellular matrix of muscle via the dystrophin-associated protein complex (DAPC).^11^^,^^12^ The DAPC is critical for resilience of muscle against contraction-induced damage/stress.^13^^,^^14^ Mutations in *DMD* that prevent expression of functional dystrophin cause DMD. In the absence of dystrophin the DAPC cannot form, and myofibers accumulate damage during regular muscle contractions. Moreover, essential molecular pathways, such as nNOS signaling, are interrupted.^11^^,^^12^^,^^13^^,^^14^ These events conclude in widespread muscle necrosis. Fibrotic and adipose tissue then infiltrate and replace the degenerated muscle tissue, leading to gradual weakening of the muscle group.^11^ While thousands of unique mutations have been identified in DMD patients, approximately 70% are exonic deletions, which represent an enormous segment of the population.^15^^,^^16^ The vast majority of DMD mutations occur in two hotspot regions at exons 2–20 and exons 45–55 in the *DMD* gene.^15^^,^^16^

To investigate therapeutic avenues applicable to the majority of DMD patients, our lab and others previously generated several mouse models recapitulating DMD deletion mutations. We recently published the generation and characterization of a multiexonic 52–54 *Dmd* deletion (Δ52–54) mouse, which recapitulates the genomic architecture of a DMD patient.^17^ Δ52–54 mice lack dystrophin because of disruption of the open reading frame (ORF) in exon 55. Hallmarks of DMD, such as elevated fibrosis in muscle tissue, progressive muscle degeneration, impaired motor function, and elevated serum creatine kinase (CK) are readily observed in the Δ52–54 mouse.^17^ Most notably, the Δ52–54 mouse model exhibits prominent early-onset cardiac hypertrophy and tachycardia.^17^ It is important to note that, while these are symptoms commonly presented by DMD patients, the cardiac phenotype of Δ52–54 mice does not manifest as dilated cardiomyopathy but instead hypertrophic cardiomyopathy.^17^ Nonetheless, the early onset of cardiac dysfunction is a novel feature with immense utility for investigating the efficacy of therapeutics.

One of the most explored therapeutic approaches for treating DMD-causing deletions is exon skipping, which removes the frameshifted exon to restore expression of a shorter, partially functional dystrophin protein.^18^^,^^19^^,^^20^ This essentially converts the DMD phenotype into a Becker muscular dystrophy (BMD)-like one. BMD is more variable, but patients typically exhibit milder symptoms than DMD patients, mostly because of in-frame deletions that permit expression of a partially functional dystrophin protein.

Antisense oligonucleotides (AONs) are a clinically validated strategy for exon skipping in DMD patients.^11^^,^^21^ AONs mask the chosen exon from the splicing machinery, resulting in its exclusion from the final mature transcript. The efficacy of AONs has been demonstrated to marginally restore production of a truncated dystrophin protein for various DMD mutations, but the effect was enough to improve clinically relevant outcomes.^21^^,^^22^ Presently, eteplirsen, golodirsen, viltolarsen, and, most recently, casimersen have been approved by the US Food and Drug Administration (FDA) for clinical use. However, AONs are limited by their transient nature, requiring regular re-administration, and current inability to be delivered to the heart via systemic delivery.^21^^,^^23^^,^^24^ Thus, while AONs may be able to lessen the disease burden of DMD to some degree, they are unlikely to stave off cardiomyopathy by current delivery methods.

Another therapeutic strategy with several clinical trials underway and more currently enrolling is micro- or mini-dystrophin gene therapy.^11^^,^^25^^,^^26^^,^^27^ Here the dystrophin coding sequencing is truncated to its most essential elements to enable its most essential functions while fitting within the limited packaging size of adeno-associated viruses (AAVs). As with exon skipping, the objective is to convert the DMD phenotype to a BMD-like one, with preliminary clinical trial results demonstrating widespread levels of dystrophin expression and possible improvements to muscle function.^26^^,^^27^ While promising, the transgene will remain as an episome, which will likely be lost due to muscle turnover, thus reducing the therapy’s efficacy over time.

With the discovery of CRISPR-Cas9 as a genome editing tool, advancements have progressed rapidly, and in less than a decade, the first therapies utilizing this technology have been approved for treating human genetic disease.^28^ The main advantage of genome editing is the potential for permanent correction of genetic mutations, thus addressing the primary cause of the disease rather than its symptoms. Exon skipping can be achieved with CRISPR-Cas9 via two approaches: (1) paired, flanking single-guide RNAs (sgRNAs) that excise the out-of-frame exon and (2) a solitary sgRNA approach harnessing the non-homologous end joining (NHEJ) DNA repair pathway, primarily used by post-mitotic cells such as myofibers, when repairing the double-strand breaks induced by Cas9.^20^ NHEJ repair typically results in random insertions and deletions (indels) that can disrupt sequences at and immediately adjacent to the Cas9 cut site. By targeting the splice donor or acceptor site of the out-of-frame exon with an sgRNA, the resulting indels from NHEJ can sufficiently disrupt these critical sequences to permanently exclude the exon from the *DMD* transcript.^20^ These exon skipping strategies have significantly restored the dystrophin protein in several *in vivo* pre-clinical DMD studies using viral and non-viral delivery methods.^20^ The most common approach evaluated is the use of paired guides for excision of the mutated exon in the *mdx* mouse model.^20^

To treat our Δ52–54 mice, we employed AAV9s packaged with *Staphylococcus aureus* Cas9 (SaCas9) and accompanying sgRNAs, systemically delivered via the temporal vein into Δ52–54 neonates, to exclude exon 55 from the final *Dmd* transcript and restore the ORF. We compared the efficacy of three different approaches. First, a pair of sgRNAs flanking exon 55 were used to excise the exon in its entirety (dual guide approach). The second utilized a solitary sgRNA targeting the exon 55 splice donor site to enable exon skipping (single guide approach). Finally, we increased the sgRNA dosage from the single guide approach relative to the SaCas9 (high guide approach). We demonstrated that our dual guide approach resulted in partial recovery of dystrophin expression, notably in the heart. Moreover, a solitary guide at a higher stoichiometry restored dystrophin in the heart at levels sufficient to prevent early-onset cardiac dysfunction in Δ52–54 mice*.* However, our single guide and high guide strategies did not significantly increase dystrophin levels in the heart. Our results reveal that single-cut exon skipping is a potential therapeutic avenue for treating the cardiac phenotype, which is the current leading cause of death in DMD patients.

## Results

### The dual guide approach can excise exon 55 and restore dystrophin expression in Δ52–54 mice

**Figure 1:**
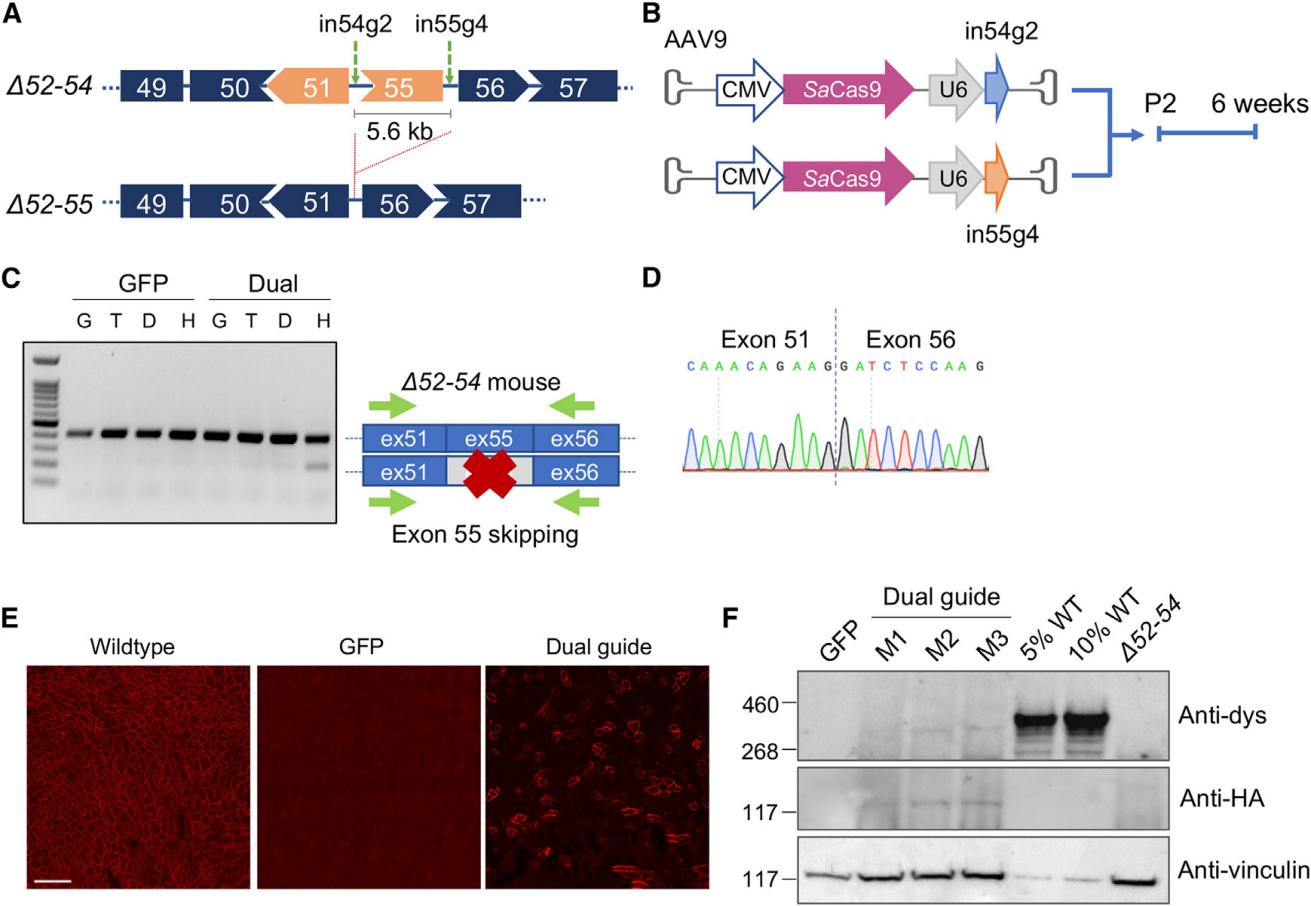
*In vivo* removal of exon 55 using a dual guide strategy in *Dmd* Δ52–54 mice restores dystrophin expression in the heart (A) Schematic of sgRNAs, in54g2 and in55g4, designed to remove a region of 5.6 kb in *Dmd* Δ52–54 that encompasses exon 55. Δ52–54 mice carry an out-of-frame deletion (out-of-frame exons in orange), and removal of exon 55 will convert the *Dmd* Δ52–54 to the *Dmd* Δ52–55 in-frame deletion. (B) The two sgRNAs (blue and orange arrows) and SaCas9 were packaged into AAVs and injected systemically via the temporal vein. Mice were analyzed 6 weeks post injection. (C) RT-PCR of cDNA synthesized from gastrocnemius (G), triceps (T), diaphragm (D), and heart (H) tissues of mice treated with either GFP or the dual guide strategy. (D) Sanger sequencing of an edited amplicon from RT-PCR derived from heart tissue. (E) Immunofluorescence staining of heart tissue detected dystrophin-positive fibers after dual guide treatment. Scale bar, 100 μm. (F) Western blot of protein from heart tissues of GFP and dual-guide-treated mice (M1, M2, M3), probing for the presence of dystrophin (anti-dys), SaCas9 (anti-HA), and vinculin (anti-vinculin).

In Δ52–54 mice, splicing of exon 51 to exon 55 disrupts the ORF and introduces a premature stop codon whereas splicing between exons 51 and 56 does not.^17^ Thus, excluding exon 55 from the mature *Dmd* transcript can restore the ORF and produce a truncated but functional dystrophin protein, converting DMD to BMD. A strategy was devised to employ SaCas9 and a pair of intronic targeting sgRNAs flanking exon 55 to excise this entire coding sequence from the genome (Figure 1A). *In vivo* delivery of SaCas9 and sgRNAs was accomplished with a pair of AAV9 vectors. Each sgRNA, driven by a human U6 RNA polymerase III promoter, was packaged into a separate AAV9 alongside a copy of SaCas9 expressed from the constitutively active cytomegalovirus (CMV) promoter (Figure 1B). Both AAV9s were administered systemically via temporal vein injection into post-natal day 2 (P2) Δ52–54 neonates, with a GFP-packaged AAV9 serving as a negative control. Six weeks post injection, muscle tissues were collected for analysis.

To evaluate the efficiency of excision of the 5.6-kb fragment encompassing exon 55, we employed a digital droplet PCR (ddPCR) assay quantifying the presence of the anticipated deletion junction. Detection of the deletion junction resulting from SaCas9 cleavage demonstrated an average editing efficiency of 0.75% at the genomic level among the four treated mice (Figure S1).

To evaluate editing at the transcript level, RT-PCR was performed on cDNA derived from the gastrocnemius, triceps, diaphragm, and cardiac tissues of GFP and dual-guide-treated Δ52–54 mice. Amplification of the region between exon 51 and exon 56 enabled edited transcripts to be discerned from the wild type by a reduction in amplicon size from 420 bp to 230 bp. Exon 55-null transcripts were primarily restricted to the heart, and Sanger sequencing validated the presence of the desired deletion (Figures 1C and 1D). Quantification by qRT-PCR concluded that, on average, among all treated mice, 11% of transcripts in the heart were edited correctly (Figure S2). Immunofluorescence staining and western blot analysis confirmed the expression of dystrophin protein in cardiac tissue, albeit at relatively sparse levels (Figures 1E and 1F). While this dual sgRNA strategy successfully excised exon 55, overall editing efficiency and dystrophin recovery were notably low and restricted to cardiac tissue.

### Skipping of exon 55 in Δ52–54 mice by a single guide approach targeting the splice donor enhances dystrophin recovery

**Figure 2:**
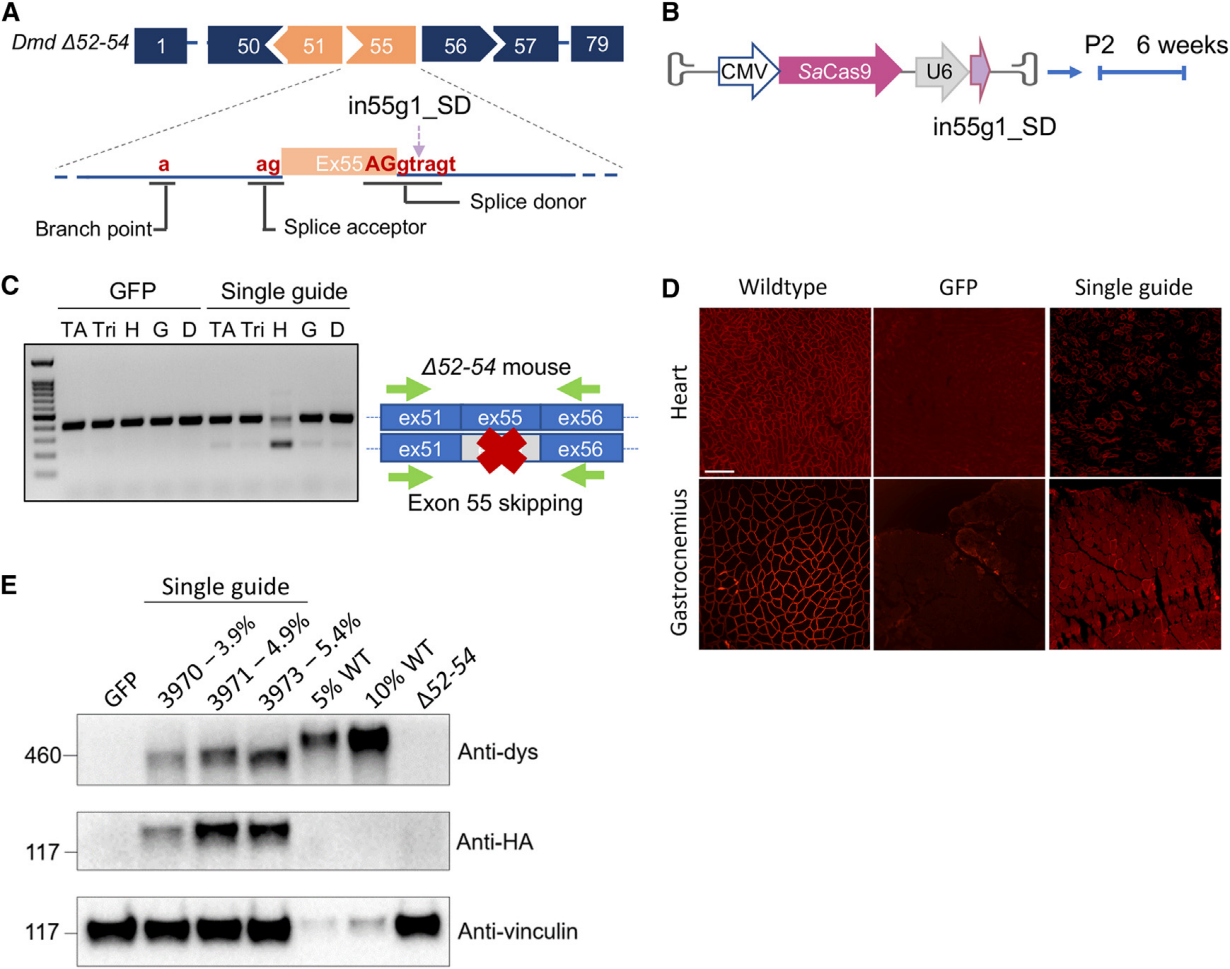
Single guide removal of exon 55 in *Dmd* Δ52–54 mice enhanced editing efficiency in the heart compared with the dual guide strategy (A) A single guide was designed to target and disrupt the exon 55 splice donor site (consensus sequence: AGgtragt; R: A or G) to enable exon 55 exclusion. (B) The plasmid expressing the in55g1_SD sgRNA (purple arrow) and SaCas9 was packaged into AAV9 and administered systemically through the temporal vein into Δ52–54 neonates. Treated mice were analyzed 6 weeks post injection. (C) RT-PCR of RNA derived from tibialis anterior (TA), triceps (Tri), heart (H), gastrocnemius (G), and diaphragm (D) tissues from GFP and single-guide-treated mice. (D) Immunofluorescence staining of heart and gastrocnemius tissues detected dystrophin-positive fibers in single-guide-treated mice. Scale bar, 100 μm. (E) Western blot probing for the presence of dystrophin (anti-dystrophin), SaCas9 (anti-HA), and vinculin (anti-vinculin) in GFP- and single-guide-treated mice. The percentage of restored dystrophin relative to wild-type (WT) levels for each treated sample is included in the lane’s label.

While successful in restoring dystrophin expression, the low editing rates observed with our dual guide strategy prompted us to devise an alternate strategy. Here a solitary sgRNA was designed to target the splice donor of exon 55. Leveraging the random indels NHEJ yields at Cas9 cut sites, we aimed to disrupt the splice donor site at the genomic level to prevent inclusion of exon 55 in the mature *Dmd* transcript. This single guide approach has been validated previously in several studies investigating CRISPR-Cas9 as a therapeutic avenue for treating DMD deletions.^29^^,^^30^^,^^31^^,^^32^^,^^33^^,^^34^^,^^35^^,^^36^ A U6-driven sgRNA, which cuts exactly in the splice donor site consensus sequence of exon 55, was packaged alongside SaCas9 expressed under a CMV promoter into a single AAV9 vector (Figures 2A and 2B).

This AAV9 was administered systemically via the temporal vein into P2 Δ52–54 neonates. An AAV9 encoding GFP was used as a negative control. Six weeks post injection, muscle tissues from these mice were collected. Genomic editing rates at the splice donor site were validated with the online Inference of CRISPR Edits (ICE) tool, using indel formation as an estimate of on-target Cas9 activity. The indel rates were 0.6%, 3.2%, 3.2%, and 8.6% for the gastrocnemius, triceps, diaphragm, and heart, respectively (Figure S3A). In the heart, all identified indels were generated in proximity to the Cas9 cut site and impacted the splice donor consensus sequence, likely prompting exon 55 exclusion (Figure S3B).

RT-PCR of cDNA derived from skeletal and cardiac muscle revealed that the largest population of edited transcripts was still in the heart; faint but detectable levels were present in the tibialis anterior, triceps, gastrocnemius, and diaphragm (Figure 2C). Compared with the dual sgRNA strategy, there was a 1.5-fold increase in edited transcripts (16% of transcripts vs. 11% with the dual guide approach) in the heart with our single sgRNA treatment, as assessed by qRT-PCR; however, this increase did not reach statistical significance (Figure S2). Dystrophin protein was again detectable by immunofluorescence in the heart and, sparsely, in the gastrocnemius (Figure 2D). Western blot analysis demonstrated an average of 4.7% protein recovery in the heart (Figure 2E). These results demonstrate that disrupting the splice donor site of exon 55 with a single guide enhanced editing efficiency and dystrophin recovery relative to a dual guide exon excision approach.

### The high guide approach to increase sgRNA dosage improves some editing outcomes for exon skipping

**Figure 3:**
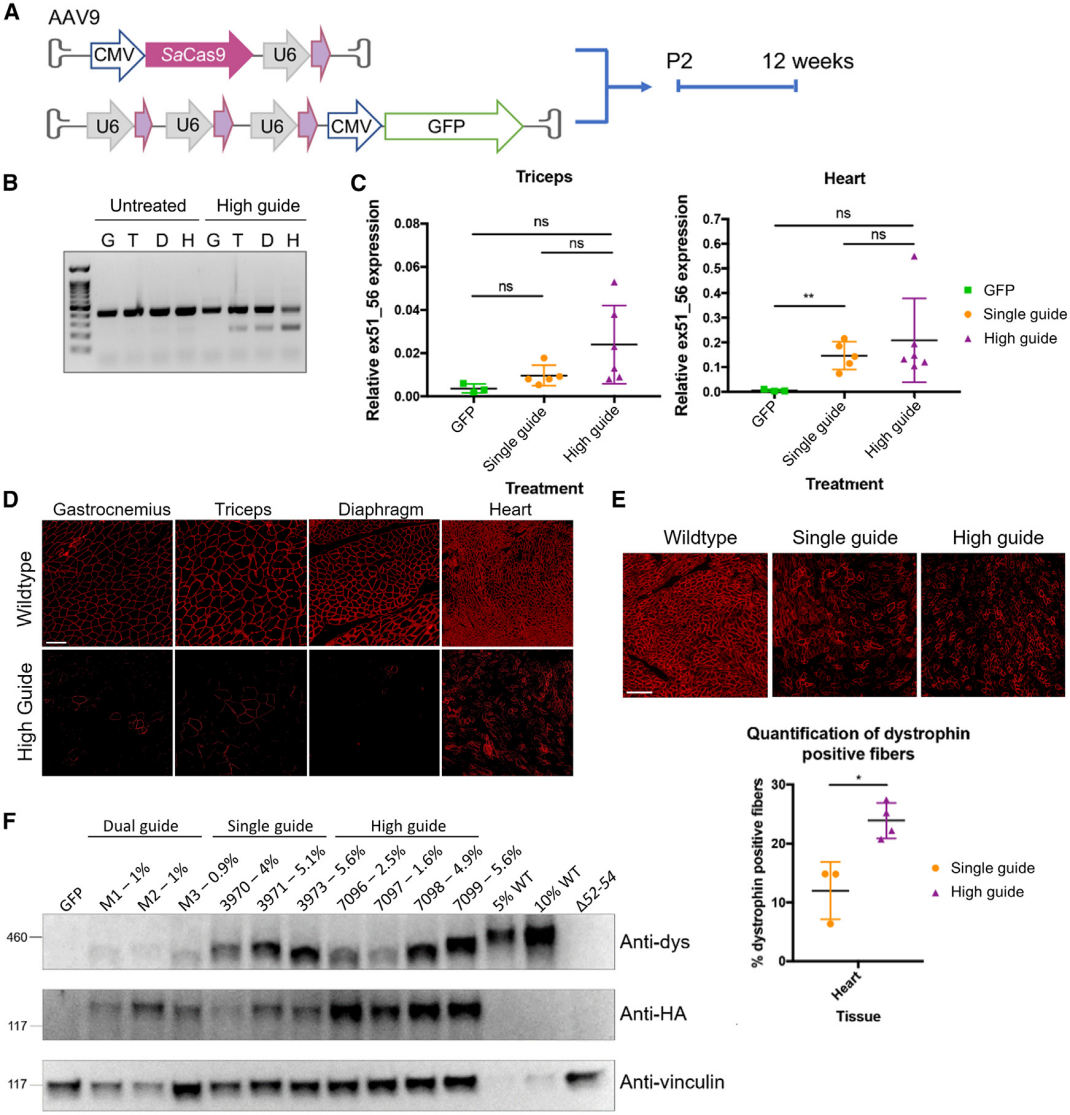
The high guide dosage approach does not significantly enhance dystrophin restoration in *Dmd* Δ52–54 mice (A) A plasmid packaging three additional copies of the in55g1_SD sgRNA was added to the single guide strategy, establishing the high guide dosage approach. Δ52-54 mice were injected systemically with the high guide dosage approach at P2 and analyzed at 12 weeks. (B) RT-PCR of cDNA of gastrocnemius (G), triceps (T), diaphragm (D), and heart (H) tissues of untreated mice and a high-guide-dosage-treated mouse. (C) The level of edited transcripts in triceps and heart tissues of GFP-, single-guide-, and high-guide-dosage-treated mice 12 weeks post injection were analyzed by qRT-PCR utilizing the expression ratio between the *Dmd* exon 51–56 junction and the WT *Dmd* transcript. (D) Immunofluorescence staining of gastrocnemius, triceps, diaphragm, and heart tissues from WT and high-guide-treated mice detected dystrophin-positive fibers. E) Immunofluorescence staining of heart tissues from WT and single-guide- and high-guide-dosage-treated mice detected dystrophin-positive fibers. Scale bar, 100 μm. Quantification demonstrated a significant increase in dystrophin-positive fibers in the high-guide-treated mice. (F) Western blot probing for dystrophin (anti-dys), SaCas9 (anti-HA), and vinculin (anti-vinculin) detected dystrophin restoration in the hearts of dual-, single-, and high-guide-treated mice. The percentage of restored dystrophin relative to levels for each treated sample is included in the lane’s label. Statistical analyses were performed with Student’s t test. ns, not significant; ∗p < 0.05, ∗∗p < 0.01.

While the single sgRNA strategy exhibited improved outcomes in the hearts of Δ52–54 mice, editing levels and recovered dystrophin remained relatively low; thus, we sought to further enhance our strategy. Work by Min et al.^29^ on CRISPR/Cas9-mediated exon skipping in *Dmd* exon 44 deletion mice demonstrated that increasing the ratio of sgRNA to Cas9 significantly augmented editing and dystrophin levels.^29^ To incorporate this strategy into our own, we employed a second AAV9 vector encoding three additional copies of the splice donor targeting sgRNA driven by three U6 promoters and an accompanying GFP stuffer sequence (Figure 3A).

P2 Δ52–54 neonates were injected with both AAV9s via the temporal vein. Two separate cohorts were further treated with a GFP-negative control and, for direct comparison, the previous single sgRNA strategy. To optimize the window for editing to occur, we collected muscle tissue 12 weeks post injection. ICE analysis concluded that the indel formation rate was 2-fold higher in the heart with the high guide approach (7.5% indels) compared with the single guide approach (3.6% indels) and that all indels impacted the splice donor sequence (Figures S4A and S4B). High-sgRNA-dosage DNA editing rates trended upward in the gastrocnemius (increase of 0.5%), triceps (increase of 2.3%), and diaphragm (increase of 1.3%) relative to the single guide strategy but did not reach statistical significance (Figure S4A).

Analysis of cDNA derived from mature mRNA unveiled exon skipping within several skeletal muscles groups as well as cardiac tissue (Figure 3B). As observed previously, the heart still harbored the largest proportion of edited transcripts, with 19.7% on average omitting exon 55 compared with 16.5% with the single sgRNA method (Figure 3C). The triceps also demonstrated an increase in edited transcripts from 1.6% to 2.4% (Figure 3C). However, the increases observed in the heart and triceps lacked statistical significance. Dystrophin-positive myofibers were observed by immunofluorescence in mice treated with the high guide strategy, although these remained sparse in skeletal muscle, with the heart notably more populated (Figure 3D). Quantification showed a 2-fold increase in dystrophin-positive myofibers in the heart with the high guide approach (24%) compared with the single guide approach (12%) (Figure 3E). Maximum levels of dystrophin protein in the hearts of treated mice were similar between the high guide approach (5.6%) and the single guide approach (5.6%), as determined by western blot (Figure 3F). Overall, augmenting the sgRNA dosage led to an increase in editing and dystrophin-positive fibers but similar dystrophin protein levels as the single guide approach, which were restricted to the heart.

### High-guide-dose-treated Δ52–54 mice show no heart dysfunction at 12 weeks of age

**Figure 4:**
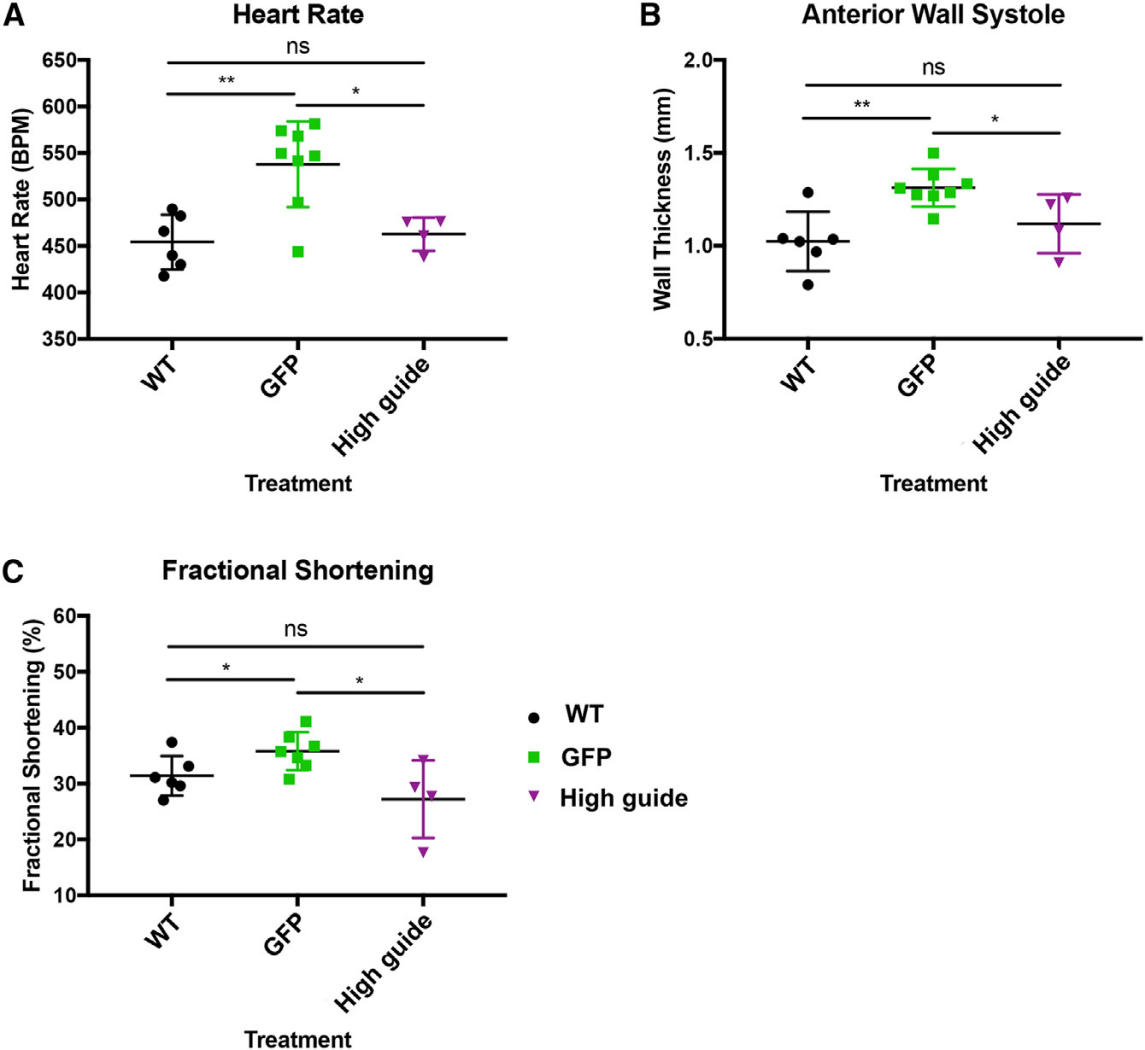
High guide dosage treatment in *Dmd* Δ52–54 mice, preventing functional decline in the heart Echocardiography was used to analyze the hearts of WT and GFP- and high-guide-dosage-treated Δ52–54 mice to determine the (A) heart rate, (B) anterior ventricular wall thickness, and (C) fractional shortening. Statistical analyses were performed with Student’s t test. ∗p < 0.05, ∗∗p < 0.01.

We proceeded to analyze disease phenotypes in Δ52–54 mice treated with the high guide approach 12 weeks after treatment administration. First, we screened cardiac phenotypes via echocardiography. Increased thickness of the left ventricular wall, suggesting cardiac hypertrophy, tachycardia, and elevated fractional shortening, characteristic of Δ52–54 mice, returned to wild-type ranges in the high-guide-treated group (Figure 4). Then, we evaluated whether the editing that occurred in skeletal muscle was sufficient to lead to any functional improvement. Forelimb/hindlimb grip strength and contractile assays revealed no difference between high-guide-dose treated Δ52–54 mice compared with GFP-treated Δ52–54 controls (Figures S5A and S5B). Serum CK levels were not significantly reduced following treatment, although they trended downward, with GFP- and high-guide-treated mice having levels 1.7- and 1.3-fold higher than wild-type mice, respectively (Figure S5C). Taken together, our results show how the high guide approach, while not improving the motor phenotype, was able to restore sufficient dystrophin expression to prevent onset of the cardiac complications associated with our Δ52–54 *Dmd* mice.

## Discussion

In this study, three CRISPR-Cas9 strategies were applied to restore truncated dystrophin expression in the Δ52 Δ52–5454 deletion mouse by skipping *Dmd* exon 55, thus restoring the ORF. We employed a dual guide, single guide, and high guide approach that showed efficacy in recovering dystrophin expression in the hearts of Δ52 Δ52–5454 mice. Moreover, we showed that the optimized high guide could prevent the early-onset cardiac phenotype characteristic of this model.

From the presented data, simplifying the delivery system and editing mechanism resulted in a boost to efficiency and dystrophin recovery. Requiring delivery of two individual but essential AAVs and two coordinated cutting events to excise exon 55 was far too inefficient at restoring dystrophin to be therapeutically impactful. Reduction to a single AAV9 and genomic target greatly improved efficacy with the single guide approach.

The high guide strategy saw an increase in edited nuclei and dystrophin transcripts in the heart because of the elevated sgRNA dosage. However, the maximal dystrophin protein recovered was similar to the single guide approach, suggesting that the increase in skipped transcripts was insufficient to notably improve the levels of expressed dystrophin protein. The on-target efficiency of our sgRNA is likely a limiting factor here, and improved dystrophin recovery may be possible with a more active sgRNA. While this strategy did reintroduce a second AAV9, only additional sgRNA copies were encoded by it. Editing and, thus, dystrophin expression would still occur with transduction of only the first AAV9 encoding the SaCas9 and sgRNA; the second AAV9 should only enhance the degree of editing by increasing sgRNA abundance. The mechanism underpinning the beneficial effect of elevated sgRNA dosage on CRISPR-Cas9 editing is not concretely understood. Min et al.^29^ utilized a dual AAV9 CRISPR-Cas9 exon skipping strategy to treat an exon 44 deletion mouse. The second AAV9 encoded three copies of their sgRNA, but they also utilized a 10-to-1 sgRNA AAV-to-Cas9 AAV ratio. Substantial increases in systemic dystrophin recovery were noted in all analyzed muscle groups. One postulation put forth suggests that Cas9 activity is enhanced in the presence of higher sgRNA levels.^29^ Another suggests that Cas9 protein translated from cytoplasmic Cas9 transcripts is capable of translocating to any nuclei in the myofiber, whereas transcribed sgRNAs are restricted to the nuclei their AAV transduced.^29^ Therefore, increasing sgRNA abundance with a separate AAV may enable sgRNA delivery to a greater proportion of myonuclei. Unbound sgRNAs are also noted to be prone to degradation, but increasing sgRNA levels may maximize their availability to complex with Cas9.^37^ A more likely reason is the preferential loss of single-stranded AAVs encoding multiple sgRNAs because this phenomenon has been published previously.^38^^,^^39^ This AAV depletion can be mitigated by employing a double-stranded self-complementary AAV (scAAV) instead.^40^ If this phenomenon is caused by specific AAV vector loss, then we likely did not see significant improvements in dystrophin restoration with our high guide treatment because we did not employ an scAAV; using scAAVs for this purpose was published after our *in vivo* experiments concluded. Additionally, we likely established a high editing floor because of delivery of the sgRNA and SaCas9 together in an AAV alongside the second AAV, unlike Min et al.^29^ where no sgRNA was packaged in the same AAV as their SpCas9. Dedicated studies are necessary to scrutinize these hypotheses and elucidate conclusions; however, the results of improved editing using increasing sgRNA dosages was observed in this study, albeit to a lesser extent than observed previously.

To the best of our knowledge, this is the first report to demonstrate single-cut CRISPR-Cas9-mediated exon skipping via intravenous delivery to neonatal DMD mice. All other therapeutic, single-cut exon skipping studies for DMD have so far relied on intraperitoneal injections for systemic delivery.^29^^,^^32^^,^^34^^,^^35^^,^^40^^,^^41^^,^^42^^,^^43^ This delivery method, while convenient, is not feasible for translation to DMD patients, and such high dystrophin restoration in various skeletal muscles is likely due to intraperitoneal delivery introducing a high concentration of AAVs to the center of mass, particularly near the diaphragm and other proximal muscle groups, which is not currently possible with intravenous routes. Injection into the murine temporal vein, however, is analogous to intravenous injection in humans, better reflecting the treatment regimens applicable to DMD patients.

None of our strategies significantly improved the motor function or skeletal muscle integrity of Δ52–54 mice because of poor editing rates and dystrophin recovery in skeletal muscle. These results align with several other studies that utilized intravenous injections to systemically deliver CRISPR-Cas9-packaged AAVs to treat DMD murine models.^34^^,^^38^^,^^44^^,^^45^^,^^46^^,^^47^^,^^48^^,^^49^^,^^50^^,^^51^^,^^52^^,^^53^ All of them evaluated a dual sgRNA approach to excise the out-of-frame exon in mature adult mice or neonates, and results were consistent across these studies. The heart typically experienced the greatest degree of editing and dystrophin restoration, whereas these outcomes ranged from minute to undetectable in skeletal muscle. Based on previous studies together with our results, it seems that, when AAV-packaged CRISPR-Cas9 genome editing therapies are delivered intravenously, the heart is the primary muscle group that is edited in mice. Only systemic administration by intraperitoneal injection has consistently demonstrated broad, high levels of dystrophin restoration across cardiac and skeletal muscle tissue. This is likely a limitation of the AAV vector, particularly AAV9, used across all of these studies, with intraperitoneal injection being more efficient than intravenous injection for systemic skeletal muscle delivery. For early efficacy studies, intraperitoneal delivery is useful because it is a less technically demanding approach and, as shown by the literature, is good at achieving high levels of skeletal muscle transduction.^29^^,^^32^^,^^35^^,^^40^^,^^41^^,^^42^^,^^43^^,^^54^ However, employing the intended clinical route of administration is strongly recommended by the FDA for preclinical development of gene and genome editing therapies.^55^ Additionally, no clinical trials employing AAVs have utilized intraperitoneal delivery.^56^^,^^57^^,^^58^^,^^59^ For studies with clinical translatability in mind, intraperitoneal delivery should be avoided and intravenous administration used instead to reflect the approach that would be considered for potential use in DMD patients.

Because our strategy could maintain wild-type heart function up to 12 weeks of age, we demonstrated that, in principle, it is feasible to prevent the onset of cardiac dysfunction present in DMD patients. However, to confirm whether the cardiac phenotype has been fully prevented or only delayed, analysis of our treatment in late stages of the disease (24–52 weeks of age) would be necessary. Improvements are also still necessary to restore sufficient dystrophin in cardiac and skeletal muscle to significantly reduce the overall disease burden of DMD.

Future work should seek to improve AAV delivery to peripheral muscles via intravenous delivery because this seems to be a major limitation of CRISPR-Cas9 therapy effectiveness for treating DMD. scAAVs have also been demonstrated to prevent depletion of sgRNA-containing AAVs in muscle and should be further evaluated for their potential to enhance dystrophin recovery. Increasing muscle tropism via chemical modifications to AAVs is another avenue to pursue. A recent study demonstrated that a dendrimer nanoparticle coating greatly enhanced systemic delivery of AAV9s to skeletal and cardiac muscle of a DMD pig model.^60^ Utilization of promoters with specificity for cardiac and skeletal muscle, such as CK8e and MHCK7, may further enhance efficacy while improving the safety profile of a DMD CRISPR-Cas9 therapy. One of the biggest leaps forward in improving AAV delivery has been from Tabebordbar et al.,^61^ who employed random mutagenesis of a motif within the AAV9 capsid protein, yielding several “MyoAAV” variants following selection. These MyoAAVs improved skeletal and cardiac muscle targeting while detargeting the liver in mice and non-human primates. When used to intravenously deliver a dual guide CRISPR-Cas9 exon skipping therapy into adult *mdx* mice, dystrophin restoration in numerous skeletal muscles was notably improved with MyoAAV over AAV9.^61^ Further increasing the sgRNA-to-Cas9 ratio by delivering a large excess of the second sgRNA-encoding AAV may additionally boost editing outcomes, as Min et al.^29^ demonstrated; however, they employed intraperitoneal delivery, and thus the efficacy of a large excess of sgRNAs delivered intravenously remains unevaluated. Increasing the number of sgRNA copies on the SaCas9-encoding AAV could further boost editing but would necessitate miniaturizing every possible feature, such as the promoters, poly(A) signal, and possibly the Cas9 because AAVs have an extremely limited packaging size of ∼4.7 kb. We additionally did not conduct off-target analysis following treatment because of the objective of this study being efficacy and not safety, with the murine sgRNAs employed not suitable for direct translation to human *DMD* sequences. This limitation of our study will need to be thoroughly addressed in any future CRISPR-Cas9 therapeutic work seeking to provide pre-clinical safety data.

Here we evaluated several approaches to exclude exon 55 from the final *Dmd* transcript in a Δ52–54 mouse model of DMD. Targeting the splice site for exon skipping with a solitary sgRNA substantially improved outcomes relative to a dual sgRNA method of excising the entire exon. Editing was notably enhanced by increasing the sgRNA dosage with a second AAV encoding multiple copies of the exon skipping sgRNA; dystrophin restoration remained similar between both single-cut strategies. While we were unable to improve the motor phenotype because of poor dystrophin recovery in skeletal muscle, sufficient dystrophin was restored in the heart to prevent early-onset heart dysfunction of Δ52–54 mice after 12 weeks. This work adds to the growing compendium of knowledge on CRISPR-Cas9 strategies for correcting DMD deletions. In this study, we demonstrate application of single-cut exon skipping delivered via a clinically relevant intravenous route into *Dmd* deletion neonates possessing an early-onset cardiac phenotype. From our findings, we anticipate that CRISPR-Cas9 could be beneficial for preventing and/or mitigating heart dysfunction in DMD patients.

## Materials and methods

### sgRNA design and cloning

*S. aureus* sgRNAs were designed using the Benchling tool (Table 1). For the dual guide experiment, sgRNAs targeting intron 54 (in54g2) and intron 55 (in55g4) were chosen based on the highest specificity score (in54g2, 83.9; in55g4, 79.5) The sgRNA used for single guide and combo guide experiments was chosen because it targets the *Dmd* exon 55 splice donor site.

**Table 1:** sgRNAs designed for dual guide, single guide, and combo guide exon skipping experiments

sgRNA	PAM	Oligonucleotide	Sequence (5′→3′)
in54g2	CAGAAT	sa_in54g2s	CACCGAAAGTCAAGAAAATACAAACC
		sa_in54g2as	AAACGGTTTGTATTTTCTTGACTTTC
in55g4	CCGGGT	sa_in55g4s	CACCGTCCTAAAAGTCTTAGTGTAG
		sa_in55g4as	AAACCTACACTAAGACTTTTAGGAC
in55g1_SD	TTGAGT	sa_in55g1_SDs	CACCGATGAAACCATGGCAAGTAAG
		sa_in55g1_SDas	AAACCTTACTTGCCATGGTTTCATC

The sgRNAs were cloned into an SaCas9-expressing plasmid containing inverted terminal repeats (ITRs) for AAV packaging (pX601-AAV-CMV::NLS-SaCas9-NLS-3×HA-bGHpA; U6::BsaI-sgRNA, Addgene plasmid 61591). Oligonucleotides were synthesized by Integrated DNA Technologies. For experimental controls, Addgene plasmid 61591 was modified to exclude SaCas9 and GFP.

### AAV9 production and *in vivo* delivery

AAV9 packaging and titering was conducted by Vigene Biosciences, and viruses were stored at −80°C. AAVs packaged with SaCas9/sgRNAs or GFP were delivered systemically at 7.5 × 10^11^ genome copies (GCs) per vector for all dual, single, and combo guide experiments via the temporal vein. In brief, P1–P2 male *Dmd* Δ52-54 mice were anesthetized on ice and injected with the AAV up to 50 μL. Functional tests were performed 12 weeks post injection, and mice were euthanized by CO_2_ inhalation. Tissues were dissected, coated with OCT, and frozen in nitrogen-cooled isopentane, followed by storage at −80°C.

### Animal husbandry

All mice were housed at The Centre for Phenogenomics (TCP; Toronto, ON, Canada) under environmental regulation of a 12-h light/dark cycle with food and water provision in individual units (Techniplast). All animal procedures were conducted in compliance with the Animals for Research Act of Ontario and the Guidelines of the Canadian Council on Animal Care. Animal protocols performed at TCP were reviewed and approved by the local animal care committee.

### Grip strength tests

Forelimb and hindlimb grip strength tests were performed by TCP based on TREAT-NMD: DMD_M.2.2.001. Age-matched 12-week-old C57BL/6J and *Dmd* Δ52–54 mice were lowered over the grid of the grip strength meter (Bioseb) with the torso parallel to the grid. Forepaws and hindpaws were allowed to attach to the grid before pulling the mouse back by the tail, and the maximal grip strength value of the mouse was recorded. The test was done in triplicates, where the average grip strength value was corrected by the mouse’s body weight.

### Echocardiography

For echocardiography, male mice were scanned using the Vevo2100 ultrasound machine (VisualSonics, Toronto, ON, Canada) with a 30-MHz transducer as described previously.^62^ All mice were scanned under 1.5% isoflurane anesthesia for ∼20–30 min with careful monitoring of the body temperature to maintain it at 37°C–38°C (TREAT-NMD: DMD_M.2.2.003).

### *In vivo* contraction assay

*In vivo* contraction tests were performed as described previously.^63^ Briefly, contractile activity was measured using the 1300A 3-in-1 Whole Animal System and analyzed using the Dynamic Muscle Control/Analysis 5.5 and 5.3 high-throughput software (Aurora Scientific). The mice were anesthetized with ketamine-xylazine solution at 100 mg/kg and 10 mg/kg to body weight, respectively, through intraperitoneal injection. Percutaneous electrodes were placed in the tibialis anterior and contractile output was measured. Specific tetanic force at 200 Hz was recorded and normalized to body weight.

### Genomic DNA isolation

Genomic DNA was isolated using the DNeasy Blood and Tissue Kit (QIAGEN) according to the manufacturer’s protocol.

### ddPCR

Removal of the 5.6-kb fragment using the dual guide approach was quantified using ddPCR with the QX200 system (Bio-Rad), which was performed at TCAG at the Hospital for Sick Children according to the manufacturer’s protocol. The junction resulting from successful deletion and *Dmd* exon 51 was amplified to quantify either the presence of editing or the wild-type allele, respectively. TaqMan hydrolysis probes labeled with fluorescein amidites (FAM) and hexachloro-fluorescein (HEX) were designed to target the deletion junction and exon 51, respectively. All oligonucleotides and probe sequences can be found in Table 2. To determine the level of editing, droplets with FAM fluorescence were quantified and normalized to the total amount of droplets. A g-block sequence (sequence included below) spanning the intron 54-to-intron 55 junction and wild-type C57BL/6 female mouse DNA were used as controls for the dual junction and exon 51 probes, respectively.

**Table 2:** Oligonucleotides utilized for molecular analyses

Experiment	Primer	Sequence (5′→3′)
Genotyping *Dmd* Δ52–54	m_in51-2-F	AGTACCATTGTCCCATATGTACATG
	m_in54-3-R	GAGTGTCCTAGAAGAAAATTTGGAATTTG
ICE analysis	sa_ex55g1_fw	GAGGCTGCTTTGGAAGAAACTCATAG
	m_sa_in55g1_rv	TTTACTGCCTCTGCCTCTTTTCTTC
RT-PCR (characterization and exon skipping)	mus_Dmd_ex51-F	CTAGAAATGCCATCTTCTTTGCTGTTG
	mus_Dmd_ex56-R	TGGCCATTTTCATCAAGATTGTGATAG
qRT-PCR (quantification of edited transcripts)	mus_Dmd_ex51-F2	TGGGTGATCTGGAAGACATCAATG
	mus_Dmd_ex51_56-R	CTCCTTGGAGATCCTTCTGTTTGATG
	ex 19 mus cdna_F	AAAAGTCAATGCCATAGCACGAG
	ex 19 mus cdna_R	CATTAACACCCTCATTTGCCATC
ddPCR (dual guide exon skipping)	ddpcr_m_in54_Fw	GAAGCCATGTTGCAAAAGTATG
	ddpcr_m_in55_rv	GCAGGCCTTGAACTCAGAAA
	Dual junction probe	TGAGGAGCCAGGGTC
	Exon 51 probe	TACCTGCACTGGCAGAC

### G-block sequence

The g-block sequence was as follows: 5′-GGCAGAAGTAGAAGCCATGTTGCAAAAGTATGACTGTTAACAATTGGCCCCACATGACTCCAAATGAGGAGCCAGGGTCACGCATAGGATCATTCTGAGTTAGCCGGGTGTTGGTGGCGCACTCCTTTAATCCCAGCACTCAGGAGGCAGAGGCAGGCGGATTTCTGAGTTCAAGGCCTGCCTGGTCTACAAAGTGAGTTCCAGGACAGCCAGGGATACACTGAGAAACCCTGTCTCAAAAAAAACAAAACAAAACAAAAACAAAAACAAAACAAAACAAAAGTCTTAGTATAAAGTGACGAGTGGATATGTTGTTGTTGTTGTTGTTATTGTTGTTGTTGTTAAGGGTGTTGTTCATAAACAGATTCTTTAATTGTTAGCCAGTTCATTATTTGTAGGGGCCAAATACAACACATGTGTGGAGAAAAAAGGAGGACCTTGGGTGCTGGTCCATGCCTAACCATATTACTTATGGTAGGAAATCTCTGCTGCTGCTTGCC-3′.

### ICE

CRISPR-Cas9 editing using the single and combo guide strategies was evaluated using the online ICE CRISPR analysis tool (Synthego). The 337-bp region encompassing the in55g1_SD-mediated cleavage site was amplified using the primers indicated in Table 2 using tissue-specific DNA from treated and untreated *Dmd* Δ52–54 mice. The amplicons were purified using the QIAquick PCR Purification Kit (QIAGEN) and sequenced with Sanger sequencing using the sa_ex55g1_fw primer. Sanger sequencing data from treated mice and untreated mice (control) were uploaded into ICE, which determined the frequency and nature of indel formation.

### RT-PCR

For RNA isolation, mouse tissues were sectioned in 30-μm slices and collected in 1.4-mm zirconium bead pre-filled tubes (OPS Diagnostics) and homogenized using a MagNA Lyser (Roche Diagnostic) for two 20-s cycles at 7,000 rpm with 3-min incubation on ice between cycles. TRIzol chloroform (Thermo Fisher Scientific) RNA extraction was conducted on the homogenized tissue. One microgram of RNA with random hexamers was used for cDNA synthesis using the SuperScript III First-Strand Synthesis System (Thermo Fisher Scientific). The cDNA was used for subsequent RT-PCR experiments using the primers in Table 2 to detect unedited and edited transcripts after exon skipping.

### qRT-PCR

qRT-PCR was performed using PowerUp SYBR Green Master Mix (Thermo Fisher Scientific) on a QuantStudio 3 system (Bio-Rad) (Table 2). All samples were run in triplicate, and data were analyzed using QuantStudio analysis software (Bio-Rad). Edited transcripts were identified using mus_Dmd_ex51-F2 and mus_Dmd_ex51_56-R primers, which only amplified when *Dmd* exon 55 was absent and exon 51 was joined to exon 56. Edited transcripts were normalized to the amount of *Dmd* transcripts expressed using the primers ex 19 mus cdna_F and ex 19 mus cdna_R.

### Western blot

Protein was extracted from homogenized mouse tissue by adding a 1:1 part solution of radioimmunoprecipitation assay (RIPA) homogenizing buffer (50 mM Tris HCl [pH 7.4], 150 nM NaCl, 1 mM EDTA) and RIPA double detergent buffer (2% deoxycholate, 2% NP-40, 2% Triton X-100 in RIPA homogenizing buffer) supplemented with protease inhibitor cocktail (Roche) as described previously.^63^ Total protein concentration was quantified using the Pierce BCA protein assay kit (Thermo Fisher Scientific). 15 μg of protein was prepared, and western blotting was conducted according to the NuPAGE electrophoresis system (Thermo Fisher Scientific) using NuPAGE 3% to 8% Tris acetate 1.5-mm mini protein gels. Dry blotting was performed using the iBlot 2 dry blotting system (Thermo Fisher Scientific). Primary antibodies utilized, all diluted in 5% milk, were mouse monoclonal anti-dystrophin (MANDYS8, Sigma-Aldrich, 1:1,000), mouse monoclonal anti-hemagglutinin (HA) (ab130275, Abcam, 1:1,000), and mouse monoclonal anti-vinculin (V284, Millipore, 1:2,500). Secondary antibody staining was conducted using a goat anti-mouse immunoglobulin H (IgG conjugated with horseradish peroxidase (HRP) (ab205719, Abcam, 1:10,000 for dystrophin and HA, 1:5,000 for vinculin). Imaging was conducted by application of the SuperSignal West Pico PLUS chemiluminescent substrate (Thermo Fisher Scientific), followed by signal detection on a Bio-Rad ChemiDoc MP imaging system. Band densitometry using ImageLab was employed to quantify dystrophin protein expression normalized to vinculin.

### Clinical chemistry

Mice were euthanized using cervical dislocation, and whole blood was collected into tubes from the chest cavity immediately after heart dissection. Blood was centrifuged at 10,000 × *g* at 4°C for 5 min. Clear serum was extracted and stored at −80°C. Serum was measured using the Liquid Creatine Kinase Reagent Kit (Pointe Scientific) according to the manufacturer’s protocol. In brief, serum was diluted in 1× PBS at a 1:4 ratio and incubated with the reagent for 2 min. Absorbance was measured at 340 nm, and readings were recorded every 2 min two more times. Final serum CK was calculated based on the manufacturer’s protocol, and serum CK was plotted relative to an average of serum CK levels of seven wild-type mice.

### Immunofluorescence staining

All muscle tissues were sectioned at 8 μm for immunofluorescence staining. Sections were fixed in ice-cold methanol and blocked with blocking buffer (3% normal goat serum, 0.2% BSA in PBS). Primary antibodies were incubated overnight at 4°C in a humidity chamber. Primary antibodies used were rabbit polyclonal anti-dystrophin (abcam15277, Abcam, 1:200) and rat monoclonal anti-Laminin-2 (α2 chain) (4H8-2, Sigma-Aldrich, 1:500). Secondary antibodies used were goat polyclonal anti-rabbit Alexa Fluor 594 (Thermo Fisher Scientific, 1:250) and goat polyclonal anti-rat Alexa Fluor 488 (Thermo Fisher Scientific, 1:250). All sections were mounted with ProLong Gold Antifade Mountant (Thermo Fisher Scientific). Sections were scanned using the 3DH panoramic slide scanner at the imaging facility at the Hospital for Sick Children, and images were acquired with CaseViewer (3DHISTECH).

### Statistical analysis

GraphPad Prism v.7 was used to conduct Student’s t test for all statistical analyses.

## Data Availability

All original data are available from the authors without any restrictions.

## Supplemental information

Document S1. Figures S1–S5

Document S2. Article plus supplemental information
