# Supplementary material for: Prevention of early-onset cardiomyopathy in Dmd exon 52–54 deletion mice by CRISPR-Cas9-mediated exon skipping
Source: Mol Ther Methods Clin Dev. 2023 Jul 17;30:246–58. doi: 10.1016/j.omtm.2023.07.004 (PMC10403712; doi:10.1016/j.omtm.2023.07.004)
Supplement: Document S1. Figures S1–S5 [file mmc1.pdf]

OMTM, Volume *30*

Supplemental information

Prevention of early-onset cardiomyopathy in *Dmd* exon 52–54 deletion mice by CRISPR-Cas9-mediated exon skipping

Matthew Rok, Tatianna Wai Ying Wong, Eleonora Maino, Abdalla Ahmed, Grace Yang, Elzbieta Hyatt, Kyle Lindsay, Sina Fatehi, Ryan Marks, Paul Delgado-Olguín, Evgueni A. Ivakine, and Ronald D. Cohn

A

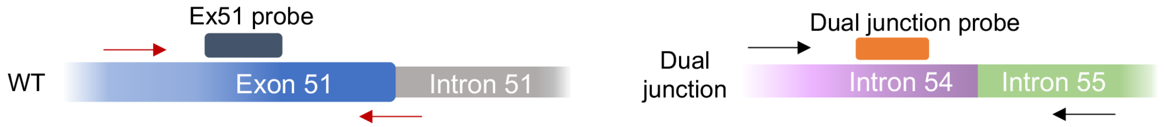

B

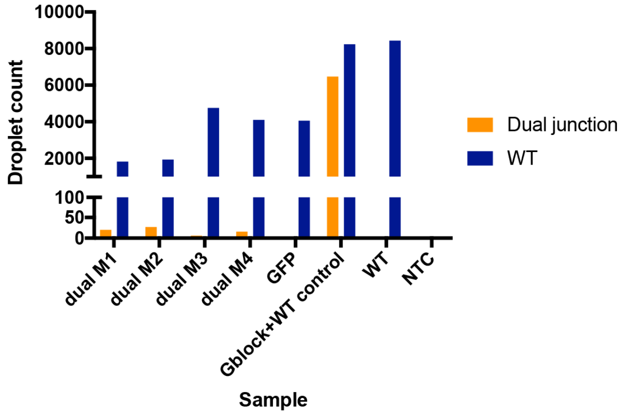

**Sample**	**Mouse**	**% editing (based on copies)**
Dual guide	Dual M1	1.08%
	Dual M2	1.38%
	Dual M3	0.13%
	Dual M4	0.39%
GFP	GFP M1	0%
ddPCR controls	½ gblock ½ B6 female	43.97%
	B6 control (female)	0%
	No template control	0%

Figure S1. Quantification of *in vivo* editing efficiency using the dual guide approach. A) ddPCR using Taqman probes was conducted to quantify the level of successful editing. Regions from the unedited allele (primers in red) and from the edited allele (primers in black) were amplified and probes labelled with either HEX or FAM fluorophores were designed to target either exon 51 (probe in blue) or the dual junction (probe in orange) region respectively. **B)** The level of editing using the dual guide approach was quantified using ddPCR.

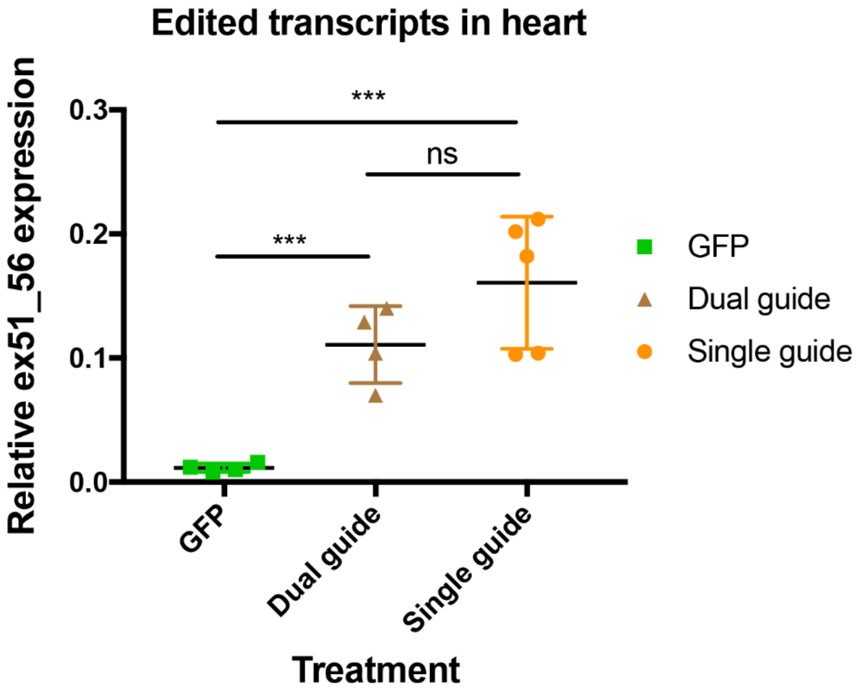

Figure S2. Quantification of editing efficiency using the single guide and dual guide approaches. The level of edited transcripts in GFP, dual guide, and single guide treated Δ52-54 mice were quantified by qRT-PCR utilizing the expression ratio between the *Dmd* exon 51-56 junction and the wildtype *Dmd* transcript. Statistical analyses were performed with Student's t-test. ns, not significant; *** P< 0.001.

A

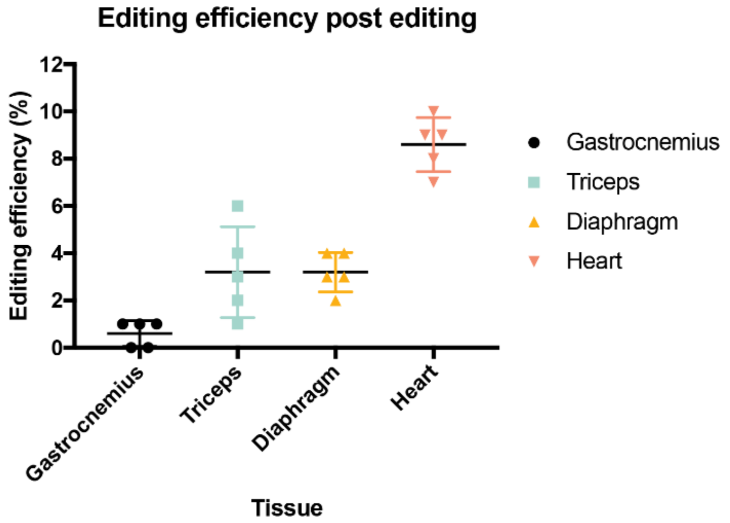

B

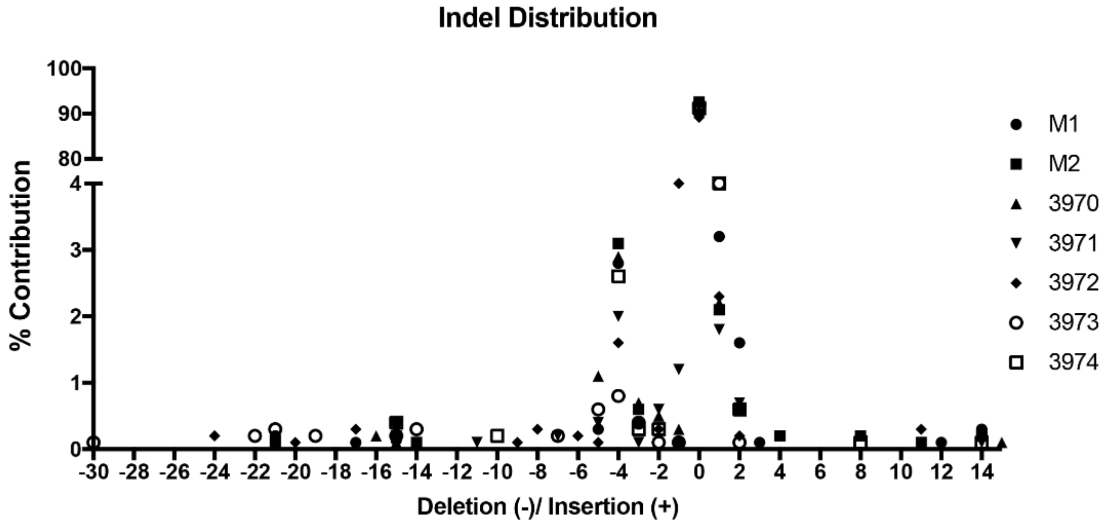

Figure S3. Analysis of genomic editing outcomes following exon 55 splice donor site targeting with the single guide approach A) Indel formation rate of several muscle groups from single guide treated Δ52-54 mice. **B)** distribution of indel sizes in the heart of dual guide (M1 and M2) and single guide (3970-3974) treated Δ52-54 mice as determined by ICE.

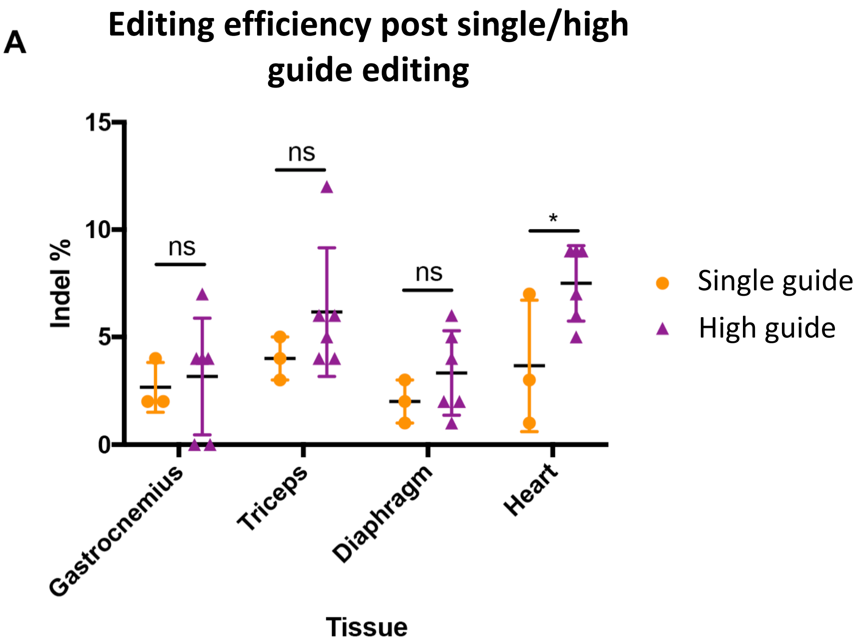

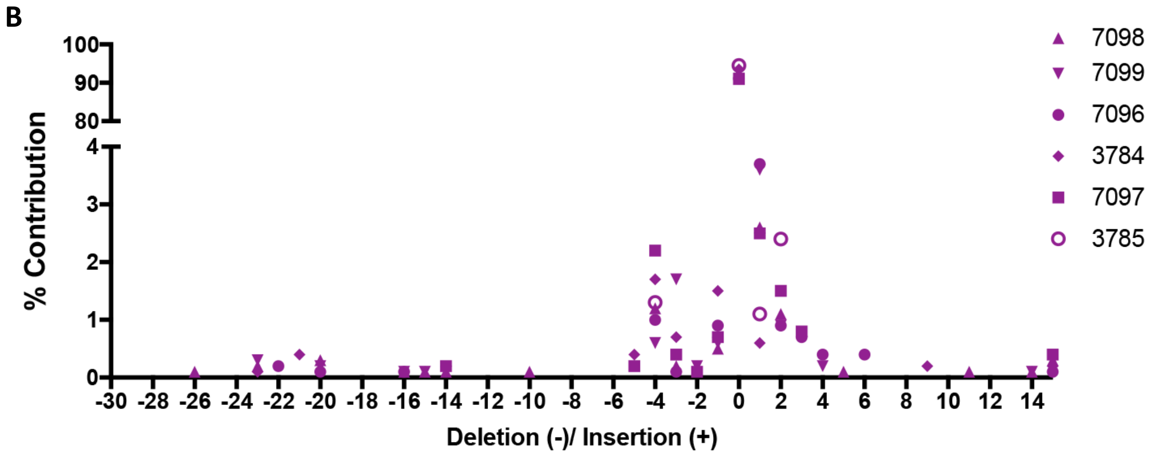

Figure S4. The high guide approach improves genomic editing. A) Editing efficiency determined by ICE analysis in gastrocnemius, triceps, diaphragm, and heart of Δ52-54 mice treated with either the single guide or high guide dosage strategies. B) Distribution of indel sizes in the heart as determined by ICE for high guide treated Δ52-54 mice. Statistical analyses were performed with Student's t-test. ns, not significant; *P < 0.05.

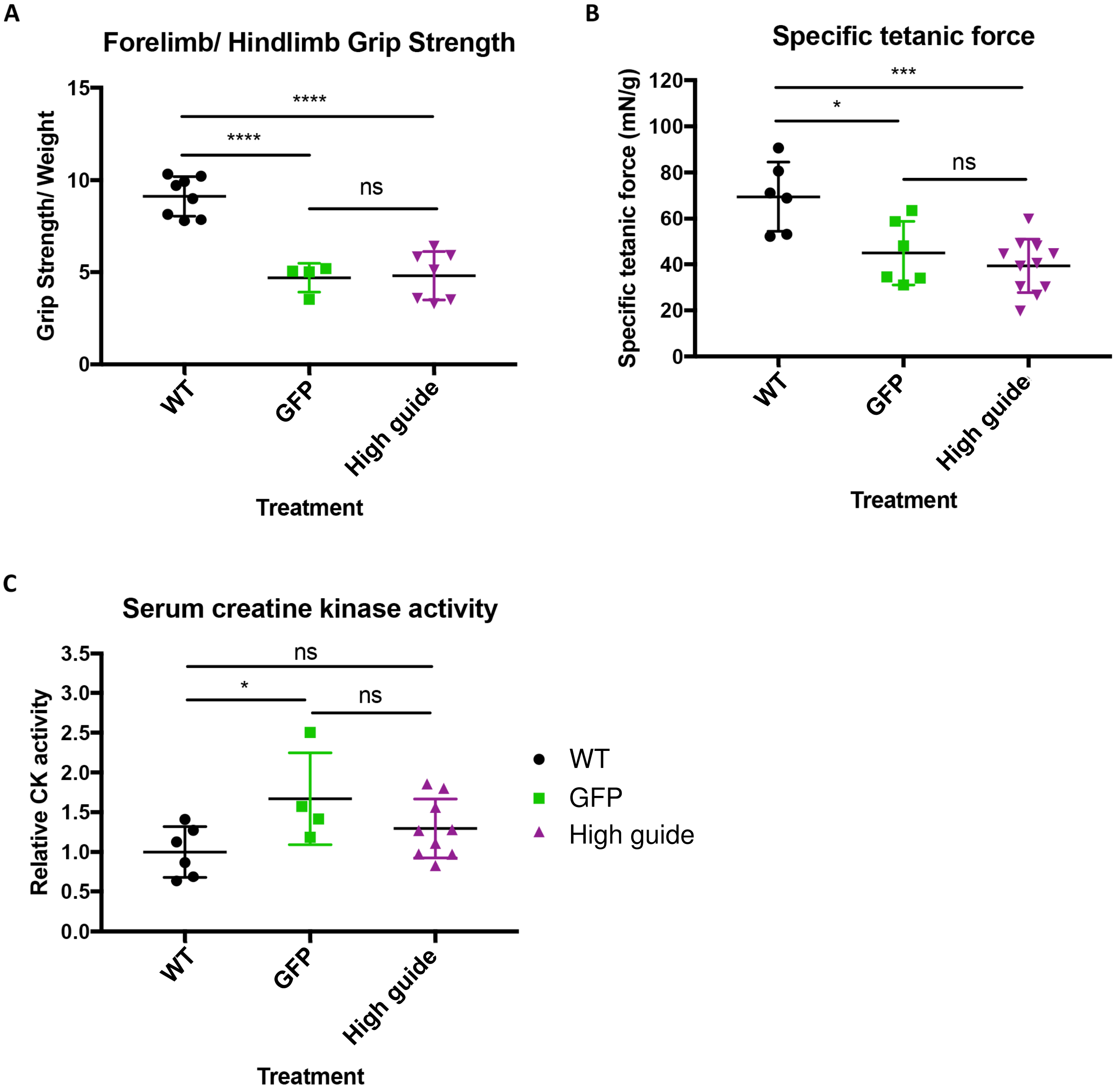

Figure S5. High guide dosage treatment in *Dmd* Δ52-54 did not improve motor function. The functionality of wildtype (WT), GFP, and High guide dosage treated mice were assessed using **A)** forelimb/hindlimb grip strength and **B)** *in vivo* contractile assay measuring specific tetanic force. **C)** Relative serum creatine kinase was evaluated in GFP and high guide dosage treated mice. Statistical analyses were performed with Student's t-test. ns, not significant; *P < 0.05; *** P < 0.001; **** P < 0.0001.
